# The Usefulness of Synovial Fluid Proteome Analysis in Orthopaedics: Focus on Osteoarthritis and Periprosthetic Joint Infections

**DOI:** 10.3390/jfmk7040097

**Published:** 2022-10-28

**Authors:** Davide Bizzoca, Lorenzo Moretti, Antonio Gnoni, Francesco Luca Moretti, Salvatore Scacco, Giuseppe Banfi, Andrea Piazzolla, Giuseppe Solarino, Biagio Moretti

**Affiliations:** 1 UOSD Spinal Surgery, Orthopaedics and Trauma Unit, Department of Basic Medical Sciences, Neurosciences and Sense Organs, AOU Consorziale Policlinico di Bari, University of Bari “Aldo Moro”, 70121 Bari, Italy; 2 Department of Basic Medical Sciences, Neuroscience and Sense Organs, Section of Clinical Biochemistry, University of Bari “Aldo Moro”, 70121 Bari, Italy; 3 National Centre for Chemicals, Cosmetic Products and Consumer Protection, National Institute of Health, 00161 Rome, Italy; 4 IRCCS Istituto Ortopedico Galeazzi, 20161 Milan, Italy

**Keywords:** synovial fluid, synovial joint, proteomics, osteoarthritis, periprosthetic joint infections

## Abstract

Synovial fluid (SF) is a viscous and mucinous substance produced by the synovium, a specialized connective tissue that lines diarthrodial joints. SF represents a source of disease-related proteins that could be used as potential biomarkers in several articular diseases. Based on these findings the study of SF has been gaining increasing importance, in recent years. This review aims to summarize the usefulness of synovial fluid in orthopaedics research and clinical practice, mainly focusing on osteoarthritis (OA) and periprosthetic joint infections (PJIs). Proteomics of the SF has shown the up-regulation of several components of the classic complement pathway in OA samples, including C1, C2, C3, C4A, C4B, C5, and C4 C4BPA, thus depicting that complement is involved in the pathogenesis of OA. Moreover, proteomics has demonstrated that some pro-inflammatory cytokines, namely IL-6, IL-8, and IL-18, have a role in OA. Several SF proteins have been studied to improve the diagnosis of PJIs, including alpha-defensin (Alpha-D), leukocyte esterase (LE), c-reactive protein (CRP), interleukin-6 (IL-6), calprotectin and presepsin. The limits and potentials of these SF biomarkers will be discussed.

## 1. Introduction

The proteome is the cell’s specific protein complement in a defined physiological context at a specific point in time [1]. Proteome analysis or “proteomics” is the large-scale protein-based systematic analysis of the proteome, or a defined sub-proteome, from a cell, tissue, or entire organism [1]. It is a relatively new discipline that has been growing rapidly in the past two decades [2]. 

Synovial fluid (SF) is a viscous and mucinous substance produced by the synovium, a specialized connective tissue that lines diarthrodial joints. Although the main function of SF is to lubricate joints, thus reducing friction between the articular surfaces, it also allows the nutrients and catabolites to circulate between the avascular articular cartilage and the vascularized synovial membrane [3,4].

SF is an ultrafiltrate of plasma, containing the same plasmatic levels of glucose and uric acid. The SF protein concentration, however, is about one-third of the plasmatic one [5]. Plasma constituents that enter joint fluid must cross a double-barrier membrane. First, the endothelial lining of the capillaries is breached, followed by movement through a matrix that surrounds synovial cells [6]. This ultrafiltrate is finally combined with the hyaluronate synthesized by the synovium [6]. It accumulates in a pathologic joint and reflects the ongoing process of the joint disorder [7].

Therefore, SF might represent a potential source of disease-related proteins that could be used as biomarkers in several articular diseases. Consequently, in recent years, the study of SF has received increasing interest within the fields of orthopaedics and rheumatology, to better investigate the pathogenesis and improve the management of several joint diseases, including osteoarthritis (OA), rheumatoid arthritis (RA), other autoimmune non-rheumatoid-arthritis conditions, osteochondrosis, articular infection, and periprosthetic joint infection (PJI) [8,9,10,11,12,13].

This review aims to summarize the usefulness of synovial fluid in orthopaedics research and clinical practice, mainly focusing on osteoarthritis and periprosthetic joint infections.

## 2. Synovial Fluid Proteome in Osteoarthritis

Osteoarthritis (OA) is the most prevalent degenerative joint disease and a leading cause of pain and disability in elderly people. The Osteoarthritis Research Society International (OARSI) defines OA as “a disorder involving movable joints characterized by cell stress and extracellular matrix degradation initiated by micro-and macro-injury that activates maladaptive repair responses including pro-inflammatory pathways of innate immunity” [14].

OA, affecting 250 million individuals worldwide, mainly adults over 65 years of age, represents a significant social health problem [15].

Even if several genetic and environmental risk factors promote the development of OA, i.e., age, gender, ethnicity, body mass index (BMI), physical activity and muscle weakness, the exact pathogenesis of OA is still unclear [16,17,18,19]. Recent studies have highlighted a potential relationship between gut microbiota alterations and OA-related pain and functional limitations [17,18,19].

The anatomopathological features of OA include cartilage degeneration, intra-articular synovial inflammation and subchondral osteosclerosis [20,21,22,23]. 

Currently, the diagnosis of OA is based on physical examination, while the progression of the joint degenerative changes could be assessed with imaging techniques only [16,20]. 

Osteoarthritis causes articular pain, joint stiffness, and loss of function, thus progressively affecting the working ability and the social life of the patient [24]. Nonetheless, the onset of symptoms occurs after the irreversible joint changes have developed [15]. Therefore, arthroplasty is still the most successful procedure for the treatment of OA, with a reportedly high patient satisfaction rate [25,26,27,28,29,30,31].

Thus, the search for synovial fluid (SF) biomarkers that could anticipate the diagnosis of OA is gaining increasing importance in orthopaedics [15]. 

Proteomics of the SF has shown the upregulation of several components of the classic complement pathway in OA samples, including C1, C2, C3, C4A, C4B, C5, and C4 binding protein alpha (C4BPA), thus depicting that complement is involved in the pathogenesis of OA [1,2,7,12,32].

Corigliano et al., in a clinical study analysing the SF, obtained from 25 arthritic knees at different stages, have recently reported that the expression levels of two complement C3 peptide fragments, i.e., C3f and C3f des arg, decrease with the progression of OA degree severity [33]. These authors consequently suggest that C3f could be a useful tool for the staging of knee OA [33].

Wang et al., in an interesting in vivo study performed on mice genetically deficient in C5, C6, or CD59a, have shown that complement, and specifically the membrane attack complex (MAC)-mediated arm of complement, is critical to the development of arthritis.

Moreover, these authors have shown that, in chondrocytes of mice genetically deficient for C5, the expression of inflammatory and degradative molecules was lower than in C5-sufficient mice [7].

Based on this finding, Wang et al. hypothesized that increased SF levels of some complement proteins should cause MAC formation, with subsequent chondrocyte apoptosis and inflammatory response [7].

It should be noted that these data still have limited clinical usefulness, since it is reported that complement inhibition causes an increased risk of infection, mainly caused by Neisseria [32]. Eculizumab, an anti-C5f human monoclonal antibody FDA-approved for the treatment of paroxysmal nocturnal haemoglobinuria (PNH) could be, however, a promising tool in the treatment of OA [32,34,35].

Proteomics studies have also suggested that some pro-inflammatory cytokines, namely IL-6, IL-8, and IL-18, could have a central role in the pathogenesis of OA [36,37,38,39].

Interestingly, Livshits et al. evaluated the circulating levels of IL-6 in 908 women at recruitment and at 5-, 8-, and 15-year follow-ups [9]. All the patients also underwent an X-ray of the knee at baseline and 10- and 15-years follow-up; the X-ray findings were finally used to classify in stages, according to Kellgren and Lawrence, the severity of knee OA in the recruited patients [9]. These authors reported that circulating levels of IL-6 are significantly higher in women who develop OA, concluding that IL-6 could be a potential therapeutic target in the treatment of knee OA [9].

Wang et al., in a controlled clinical radiographic study comparing 33 patients with primary knee OA at different stages with 15 health subjects, observed that plasma, synovial fluid, and articular cartilage IL-18 levels were significantly increased in OA patients, and these elevated levels were positively correlated with radiographic OA severity [40].

### Synovial Fluid Proteome and Periprosthetic Joint Infections

Periprosthetic joint infections (PJIs) are among the leading causes of revision prosthetic surgery, accounting for 25% of failed total knee replacement (TKR) and 15% of failed total hip replacement (THR) [12,30,40,41,42,43,44,45,46,47,48].

Hip and knee PJI are currently diagnosed according to the 2018 Philadelphia ICM criteria [33,35]. Two positive cultures or a sinus tract presence are considered major criteria; at least one major criterium is sufficient to diagnose a PJI [33].

In absence of major criteria, minor criteria are considered to diagnose a PJI; in this case, a minimum score of 6 (out of 12) is needed to diagnose a PJI [33]. Minor criteria scoring is defined as follows: 2 points for a serum CRP > 1 mg/dL; 2 points for D-dimer > 860 ng/mL; 2 points for erythrocyte sedimentation rate (ESR) > 30 mm/h; 3 points for a synovial fluid white blood cell count > 3000 cells/μL; 3 points for an increased synovial fluid alpha-defensin (signal to cut-off ratio > 1); 3 points for an elevated synovial fluid leukocyte esterase (++); 3 points for polymorphonuclear percentage > 80% and 2 points for synovial CRP > 6.9 mg/L.

The search for a biomarker that, together with clinical and radiological findings, could improve the management of such a patient is currently a big challenge for orthopaedic surgeons. 

In this context, the study of synovial fluid proteome might play a central role in future research settings and daily clinical practice.

Alpha-defensin (AD) is an antimicrobial protein released by neutrophils in response to pathogens [13]. AD then enters the pathogen’s cell membrane and causes its rapid killing, thus supporting the immune system [13].

According to ICM Philadelphia 2018, an elevated synovial fluid AD, defined as the signal to cut-off ratio > 1, is a minor criterium to define PJI diagnosis, hence, in the absence of major criteria, AD should be routinely assessed to confirm or rule out a PJI [33].

Leukocyte esterase (LE) is an esterase expressed in white blood cells, thus their presence in a sample indicates leucocyte expression. An elevated synovial fluid LE is a minor criterium to define PJIs, according to 2018 ICM Philadelphia; it is a diagnostic tool with a 2+ cut-off. It is a rapid and inexpensive test [9].

Ahmad et al. [1], in a recent meta-analysis based on 42 clinical studies, demonstrated that synovial LE does not reach a diagnostic accuracy higher than positive culture bacteriology or synovial white cell count, thus limiting the clinical relevance of this biomarker. 

C-reactive protein (CRP), i.e., a pentameric protein member of the pentraxin family of proteins, is an acute-phase protein synthesized by the liver. 

Although the diagnostic utility of synovial fluid CRP concentration has been debated in recent years, according to ICM Philadelphia 2018, a synovial CRP concentration > 6.9 mg/L is a minor criterion for PJI diagnosis.

IL-6 is a pro-inflammatory cytokine that induces the expression of a variety of proteins responsible for acute inflammation [28,49].

Yu et al. [49], in a prospective study that recruited 139 patients affected by hip or knee PJI, showed synovial IL-6 has a higher diagnostic accuracy for PJI, compared with synovial fluid CRP. These authors recommend assessing synovial IL-6 in patients with an increased serum IL-6 [49].

Calprotectin is a cytoplasmatic calcium- and zinc-binding protein expressed mainly in neutrophils; it is released in the extracellular environment following neutrophil activation and exhibits anti-microbial activity [50].

Faecal CPT has been used for many years in the diagnosis of inflammatory bowel disease (IBD) [23], but in more recent years has been receiving increasing interest relative to the study and diagnosis of PJI. It is a cheap test, compared to other biomarkers, and is easy to use.

Wouthuyzen-Bakker et al. [50], conducted a pilot prospective study comparing 19 patients suffering from PJI to 42 control patients, in which the calprotectin test was considered suggestive of PJI when >50 mg/L. This cut-off level showed an excellent diagnostic accuracy for PJI, with an area under the curve of 0.94, a sensitivity of 89%, and a specificity of 90% [50].

Presepsin, i.e., the N-terminal fragment of the soluble cluster of differentiation 14-subtype (sCD14-ST), is released in circulation after activation of defence mechanisms, mainly bacterial phagocytosis [26,51,52].

This biomarker, originally studied and validated in the diagnosis and prognosis stratification of sepsis [53], has recently been proposed as a potential biomarker for the study of PJI and septic arthritis [26,51,52].

Imagine et al. [20], have recently evaluated synovial fluid and serum PS and procalcitonin (PCT) levels in 18 patients with septic arthritis (SA), compared with 28 patients affected by osteoarthritis (OA), to determine whether presepsin would be useful in the diagnosis of SA. 

These authors observed that synovial fluid, blood presepsin and blood PCT were significantly higher in the SA group than in the OA group. Synovial fluid presepsin exhibited both 100% sensitivity and 100% specificity in the SA group, which were at higher rates than those for blood presepsin and PCT. Thus, Imaga et al. concluded that synovial fluid presepsin could be a new biomarker of septic arthritis [20].

## 3. Conclusions

Synovial fluid proteome analysis is a promising approach in the study of OA and PJIs. However, further studies with larger sample sizes and matched control groups are needed to better define the limits and potentials of SF proteome in the assessment of OA and PJIs.

## Data Availability

Not applicable.
